# Discriminant Model for Insulin Resistance in Type 2 Diabetic Patients

**DOI:** 10.3390/medicina59050839

**Published:** 2023-04-26

**Authors:** Erislandis López-Galán, Rafael Barrio-Deler, Manuel Alejandro Fernández-Fernández, Yaquelin Del Toro-Delgado, Isaac Enrique Peñuela-Puente, Miguel Enrique Sánchez-Hechavarría, Mario Eugenio Muñoz-Bustos, Gustavo Alejandro Muñoz-Bustos

**Affiliations:** 1 Facultad de Medicina No.2, Universidad de Ciencias Médicas de Santiago de Cuba, Santiago de Cuba 90100, Cuba; erislandislopez@infomed.sld.cu (E.L.-G.); manu000308@gmail.com (M.A.F.-F.); yaquelindeltoro584@gmail.com (Y.D.T.-D.);; 2 Hospital Pediátrico Juan de la Cruz Martínez Maceira, Santiago de Cuba 90100, Cuba; rdeler33@gmail.com; 3 Programa de Promoción de la Salud y Prevención de la Enfermedad del Núcleo Científico Tecnológico para el Desarrollo Costero Sustentable, Facultad de Medicina, Universidad Católica de la Santísima Concepción, Concepcion 4090541, Chile; 4 Núcleo Científico de Ciencias de la Salud, Facultad de Ciencias de la Salud, Universidad Adventista de Chile, Chillán 3780000, Chile; 5 Departamento de Kinesiología, Facultad de Medicina, Universidad de Concepción, Concepción 4030000, Chile; marmunozb@udec.cl; 6 Escuela de Kinesiología, Facultad de Salud y Ciencias Sociales, Campus El Boldal, Sede Concepción, Universidad de las Américas, Santiago 4030000, Chile

**Keywords:** type 2 diabetes mellitus, insulin resistance, lipid profile, anthropometric indicators

## Abstract

*Introduction:* Patients with type 2 diabetes mellitus tend to have insulin resistance, a condition that is evaluated using expensive methods that are not easily accessible in routine clinical practice. *Objective:* To determine the anthropometric, clinical, and metabolic parameters that allow for the discrimination of type 2 diabetic patients who have insulin resistance from those who do not. *Methods:* A cross-sectional analytical observational study was carried out in 92 type 2 diabetic patients. A discriminant analysis was applied using the SPSS statistical package to establish the characteristics that differentiate type 2 diabetic patients with insulin resistance from those without it. *Results:* Most of the variables analyzed in this study have a statistically significant association with the HOMA-IR. However, only HDL-c, LDL-c, glycemia, BMI, and tobacco exposure time allow for the discrimination of type 2 diabetic patients who have insulin resistance from those who do not, considering the interaction between them. According to the absolute value of the structure matrix, the variable that contributes most to the discriminant model is HDL-c (−0.69). *Conclusions:* The association between HDL-c, LDL-c, glycemia, BMI, and tobacco exposure time allows for the discrimination of type 2 diabetic patients who have insulin resistance from those who do not. This constitutes a simple model that can be used in routine clinical practice.

## 1. Introduction

Insulin is the most important hormone for energy metabolism of glucose, fat, and protein, as well as for maintaining homeostasis [1]. Altered insulin secretion and action are major contributors to the pathogenesis of diabetes mellitus (DM) [1,2]. The lowest responsiveness (maximal effect of insulin) or sensitivity (insulin concentration required for half the maximum response) to the actions of insulin is called insulin resistance (IR) [1]. There are two types of IR: genetic and acquired. Genetic IR may include some physiopathological factors such as an alteration in insulin receptors, signal changes after combination, altered β3-adrenergic receptors, primary target cell defects, autoantibodies to insulin, accelerated insulin degradation, and mitochondrial dysfunction. Acquired IR includes an increase in the levels of anti-regulation, drug-induced hormones, glucose intoxication, glutamine enzyme, glucose transporter defect, lipid toxicity, inflammatory mechanism, and a decrease in insulin secretion rate and hormone activity in tissue [3]. In several forms of insulin resistance, reactive oxygen species have also been shown to play an important role [4]. IR is considered an independent risk factor for cardiovascular diseases and type 2 DM and is associated with arterial hypertension, dyslipidemia, and obesity. However, the presence of these conditions cannot be used as evidence for the coexistence of IR [5].

The evaluation of IR is important to determine the type of DM and to plan an optimal management and prevention strategy in clinical practice. It is also valuable in research fields such as the development of new antidiabetic drugs, experimental and clinical studies on DM, and metabolic diseases [1]. Despite this, the assessment of IR requires sophisticated methodologies that are not available for use in daily clinical practice [6]. The hyperinsulinemic-euglycemic clamp is considered the gold standard test for the evaluation of IR. It directly and precisely measures IR but has the limitation of being an invasive and slow procedure. Another method that investigates insulin sensitivity is the glucose tolerance curve, but it is also time-consuming and requires frequent blood samples [2]. The costliness and impracticality of these methods has prompted the development of new techniques for estimating insulin sensitivity through mathematical models [7].

The homeostasis model of insulin resistance (HOMA-IR) measures the level of insulin and glucose in the blood and has been widely used, especially in epidemiological and clinical studies [2,7]. Insulin, for its part, has a high intra-individual and inter-individual biological variability [4]; moreover, it is expensive and not available in most developing countries [8]. These aspects have a direct impact on the estimation of IR using the HOMA-IR index and other formulas that use the insulin value in their calculations. Attempts have been made to identify other parameters that could be useful for evaluating IR. The Triglycerides/HDL-c Index (THI) has been suggested as a useful alternative for estimating insulin action, [6] as has the Triglycerides/Glucose Index (TGI) [6,9].

Nutritional anthropometric indicators can be used as alternative methods for evaluating IR, with the advantages of low cost, ease of execution, and availability in clinical practice [10]. In the current literature, there is still no definitive consensus on the best anthropometric method for predicting IR in adults [11]. It is vitally important to incorporate other diagnostic methods, new parameters, or laboratory tests that may have a predictive value in the diagnosis of IR. The aim of this study was to demonstrate a discriminant model with anthropometric, clinical, and metabolic parameters that make it possible to discriminate type 2 diabetic patients who have insulin resistance from those who do not. Current reports indicate that there are insufficient studies that evaluate these parameters simultaneously.

## 2. Methods

A cross-sectional analytical observational study was carried out in patients with a personal pathological history of type 2 diabetes mellitus who did not have pharmacological treatment for this disease, of both sexes, older than 18 years, and belonging to the basic work group No. 2 of the Ramón López Peña Polyclinic of the Santiago de Cuba municipality. Patients with a physical limitation preventing the pertinent measurements or amputation of their upper or lower limbs; patients with a personal pathological history of cardiovascular disease in its coronary, cerebrovascular, or peripheral arterial form; and those who had chronic kidney disease or were diagnosed during the investigation were excluded from this study. The sample comprised 92 patients (27 men).

### 2.1. Information Collection

Anthropometric measurements: height (cm) and weight (Kg) were measured using a SOEHNLE Professional^®^ stadiometer scale with a precision of 0.1 cm. Waist circumference and hip circumference measurements were obtained with a non-extensible tape measure less than 1 cm wide.

Blood pressure was always taken in the morning using the eclectic method with an SKN brand aneroid sphygmomanometer, certified as fit for use and numbered 1567180.

### 2.2. Complementary Exams

Biochemical studies were conducted in the clinical laboratory of the Juan Bruno Zayas Alfonso Hospital. The blood chemistry study was conducted using a Hitachi 902 chemistry autoanalyzer from the Roche Company with HELFA^®^ brand reagents. Glycemia was spectrophotometrically determined at 500 nm using the glucose oxidase enzymatic method. Total cholesterol and triglycerides were determined via colorimetric methods using spectrophotometry. HDL-c and LDL-c were determined via enzymatic methods, and the results were reported in mmol/L. Serum insulin concentrations (μIU/mL) were determined using the electrochemiluminescence method with Hitachi Elecsys 2010 equipment from the Roche Company.

### 2.3. Preparation and Processing of Information

Statistical analysis was performed using the SPSS version 22.0 program of IBM Corp. in Armonk, NY. Kolmogorov–Smirnov and Levene’s tests were applied to determine the type of distribution that the data presented and the equality of variances, respectively. In the comparison of groups, a *t*-test of independent samples was used for variables that presented a normal distribution and equal variances, and the Mann–Whitney U test for those that did not. A multivariate analysis (discriminant analysis) was applied to establish the discriminatory explanatory power of the characteristics that differentiated type 2 diabetic patients with insulin resistance from those without it. The assumption of multivariate normality was determined, and Box’s M test was applied to verify the equality of the covariance matrices; the variables that did not comply with this assumption were eliminated from the analysis. The ability of the different variables, TGI, THI, and discriminant model to distinguish between patients with and without insulin resistance was evaluated using the ROC curve (Receiver Operating Characteristic). The area under the curve, sensitivity, specificity, and predictive values for the cut-off points determined with the Youden index were estimated precisely and via a 95% confidence interval. In addition, the Spearman rank correlation coefficient was determined to measure the association between the HOMA-IR, TGI, THI, and discriminant model.

### 2.4. Fisher’s Linear Discriminant Function

This is a statistical method used to find a linear combination of features that characterizes or separates two or more classes of objects [12]. The model is described by the following equation:

Y = a_0_ + a_1_ × X_1_ + a_2_ × X_2_ + a_3_ × X_3_ + a_4_ × X_4_ + a_5_ × X_5_

a_0_ = Constant

a_p_ = Discrimination coefficients

X_p_ = Independent variables

Bioethical considerations

This research took into account the ethical principles for medical research in humans according to the Declaration of Helsinki of the World Medical Association. All the procedures carried out correspond to the stipulations in the Regulation of the General Health Law of the MINSAP in relation to scientific research.

## 3. Results

In Table 1, the variables analyzed in this study are compared between type 2 diabetic patients with and without insulin resistance. Except for age, age at debut, systolic blood pressure, and height, all other variables had a statistically significant association with the HOMA-IR.

Table 2 shows the variables used in the discriminant model (HDL-c, LDL-c, GLY, BMI, and TET) with their respective discrimination coefficients. HDL-c presented the highest absolute value of the structure matrix.

Table 3 shows the efficacy of the discriminant model in the detection of type 2 diabetic patients with insulin resistance. The discriminant model shows a relationship between sensitivity and specificity, area under the curve, and high positive and negative predictive values.

**Figure 1:**
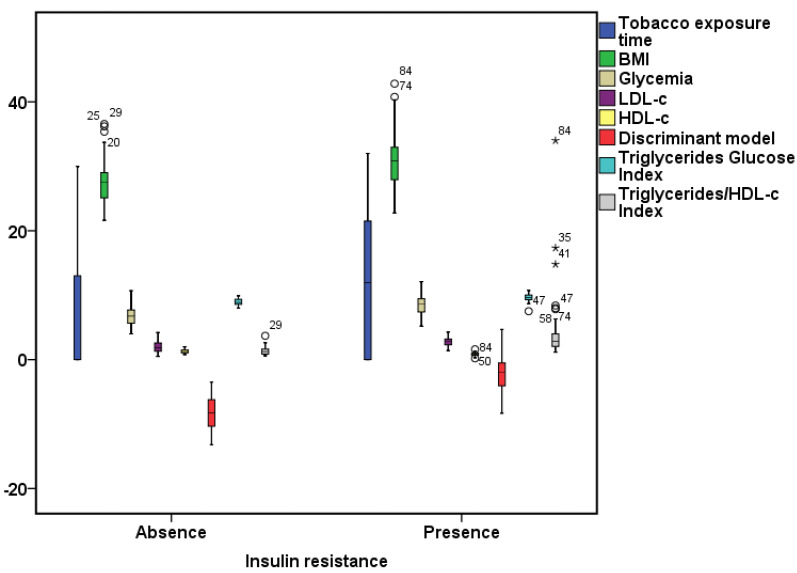
Comparison of the different variables, discriminant model, Triglycerides/Glucose Index, and Triglycerides/HDL-c Index between patients with and without insulin resistance.

**Figure 2:**
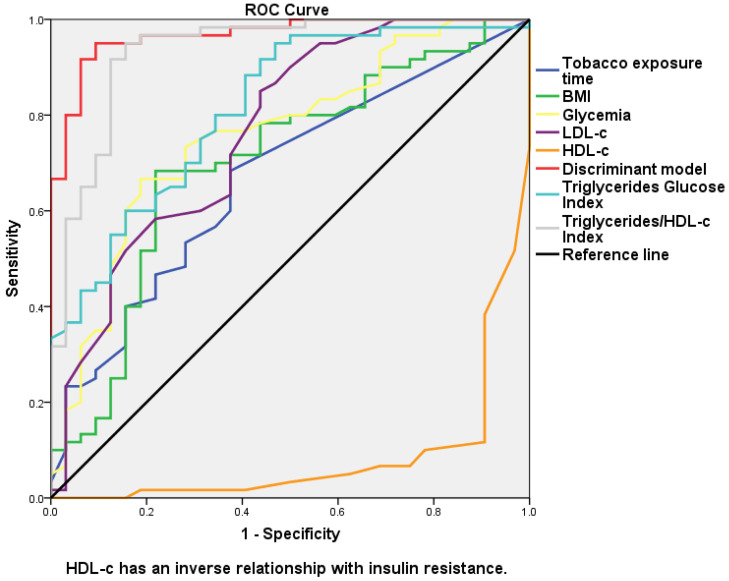
ROC curve for the different variables, discriminant model, Triglycerides/Glucose Index, and Triglycerides/HDL-c Index in order to distinguish between patients with and without insulin resistance.

**Figure 3:**
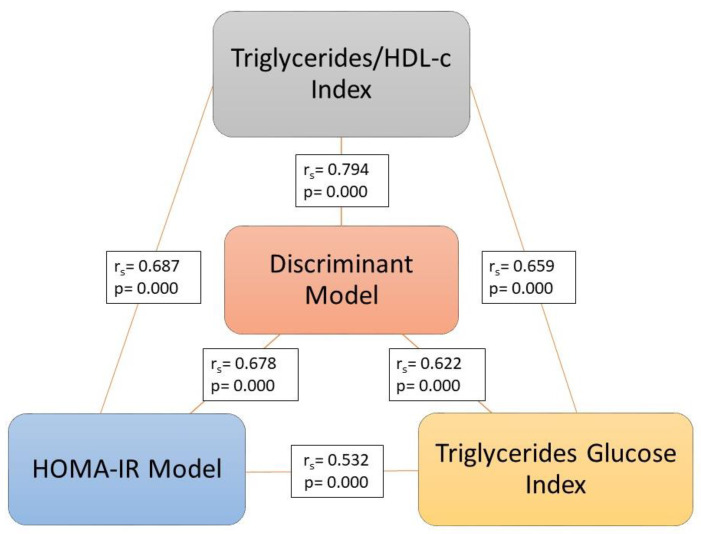
Correlation between the discriminant model, Triglycerides/Glucose Index, Triglycerides/HDL-c Index, and HOMA-IR.

The ability of the discriminant model to discriminate between type 2 diabetic patients with insulin resistance from those who do not have it was greater than the variables used separately, the Triglycerides/Glucose Index, and the Triglycerides/HDL-c Index (Figure 1 and Figure 2). The discriminant model showed a good correlation with the HOMA-IR (r_s_ = 0.678, *p* = 0.000), as well as the Triglycerides/Glucose Index (r_s_ = 0.532, *p* = 0.000) and the Triglycerides/HDL-c Index (r_s_ = 0.687, *p* = 0.000) (Figure 3).

## 4. Discussion

Most of the variables analyzed in this study had a statistically significant association with the HOMA-IR. However, only HDL-c, LDL-c, GLY, BMI, and TET make it possible to discriminate type 2 diabetic patients with insulin resistance from those who do not have it, considering the interaction between them in a combined model. The variable that contributed most to the discriminant model was HDL-c, according to the absolute value of the structure matrix.

It is well established that elevated triglycerides, reduced HDL-c, and increased small and dense LDL particles (atherogenic lipid triad) characterize the lipid profiles of diabetic patients. This dyslipidemia has been associated with visceral obesity, the presence of liver fat, and insulin resistance [13,14,15]. Alternative IR markers based on lipid measurement are useful for detecting individuals with IR when faced with the problems presented by insulin measurement and its action [6]. It has been postulated that insulin resistance induced by free fatty acids is a consequence of alterations in the transduction of the postreceptor insulin signal inside the cell. The association between lipid accumulation, anthropometric indicators, and insulin resistance is well established [16]. Adipose cell type composition, adipose mitochondrial DNA expression, and the percentage of body fat play critical roles in predicting insulin resistance [17].

Studies that have evaluated the ability of BMI to predict IR have presented conflicting results. Ascaso et al. [18], in a study on non-diabetic patients, found a correlation between BMI and IR, while Chang et al. [19] demonstrated that BMI has a weak capacity to identify IR. Other studies reported that BMI was negatively associated with insulin sensitivity assessed using different methods [20,21,22]. In the present study, the association between BMI and HOMA-IR was demonstrated and used in the model for a new discriminant function that allows the differentiation of patients who have IR from those who do not. However, although most evidence suggests a negative association between adiposity and insulin sensitivity, it is important to note that fat distribution is an important regulator of insulin sensitivity. Therefore, the different metabolic effects of different adipose depots may explain part of the variability in the association between BMI and insulin sensitivity [23]. It has been suggested that insulin resistance is more associated with visceral fat than subcutaneous fat [22]. However, in this study, despite the fact that the visceral adiposity index (VAI) was significantly associated with the HOMA-IR, it was not used in the discriminant model. In this sense, more in-depth research should be conducted on fatty deposits. For example, significant differences in insulin sensitivity have been shown in subjects with high liver fat content compared with those without, while no differences were detected between groups that had different volumes of visceral adipose tissue [24].

Tobacco consumption has a deleterious effect on diabetic patients, accelerates microvascular and macrovascular complications, and increases the risk of global and cardiovascular mortality [25]. Its systemic, vascular, and prothrombogenic effects generate endothelial dysfunction in systemic arteries, deleteriously modify the lipid profile, increase arterial stiffness [13], cause insulin resistance, and worsen the metabolic control of DM [26]. These negative effects increase with the number of cigarettes per day and with the time of exposure to tobacco [14]. This justifies the use of the TET in the discriminant model, despite being a difficult variable to obtain with precision.

The discriminant model showed a better sensitivity–specificity relationship with the area under the curve and higher positive and negative predictive values than the traditional Triglycerides/Glucose Index and Triglycerides/HDL-c Index. The capacity of the discriminant model to discriminate between patients with insulin resistance from those without it is greater than the Triglycerides/Glucose Index and the Triglycerides/HDL-c Index. The correlation with the HOMA-IR was higher in the discriminant model than in the Triglycerides/Glucose Index but lower than in the Triglycerides/HDL-c Index.

In clinical practice, the estimation of a patient’s IR from fasting insulin values using the HOMA-IR is unlikely to be accurate. The range over which insulin is measured is small, the pulsatility of insulin secretion, and the effects of stress or exercise could affect the interpretation of results [27]. The measurement of insulin concentration has some difficulties, such as the cross-reactivity of proinsulin, the type of insulin radioimmunoassay kit used [2,27], blood sampling with heparin, hemolysis, and the state of dyslipidemia. It has been reported that the error results from the probability that the proinsulin cross-reaction is greater in diabetic patients whose proinsulin concentration is usually higher. Heparin and hemolysis can reduce insulin concentration by up to 25%. In patients with dyslipidemia, the measured insulin level may be 15% higher than the actual concentration. These limitations must be carefully considered when employing the HOMA-IR method [2]. The strength of the discriminant model perhaps lies in the correlation between variables that rapidly change, as occurs with intravenous glucose, and others that are more stable over time.

As for the limitations of this study, it must be recognized that it may present a certain bias because the individuals were evaluated only once, and the intra-individual biological variability of the biochemical and clinical determinations could not be minimized. Despite the fact that insulin resistance is related to weight, lipids, and smoking, it is known that other parameters have correlations such as high-sensitivity C-reactive protein, reactive oxygen species, uric acid [4] adipokines [28], or other parameters that were not taken into account, and this should be considered when interpreting the results. It is suggested that future studies carry out investigations that use a hyperinsulinemic-euglycemic clamp and try to determine the a priori value of the discriminant model in the diagnosis of IR in patients with type 2 DM.

## 5. Conclusions

The association between HDL-c, LDL-c, glycemia, BMI, and tobacco exposure time allows for the discrimination of type 2 diabetic patients who have insulin resistance from those who do not. This constitutes a simple model that can be used in routine clinical practice.

## Figures and Tables

**Table 1:** Relationship between clinical, anthropometric, and biochemical characteristics with the presence of insulin resistance in the study population.

Variables	Presence of Insulin Resistance				*p*
	No		Yes		
	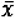	DS	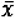	DS	
Age	50.47	10.38	51.25	8.73	0.703
Weight	74.66	10.44	83.10	13.57	0.003
Body Mass Index (BMI)	27.91	3.90	30.88	4.36	0.002
Hip Circumference	95.78	10.80	102.57	10.82	0.005
Waist–Hip Index	0.956	0.106	1.01	0.11	0.018
Waist–Height Index	0.559	0.077	0.636	0.092	0.000
Body Adiposity index	28.06	6988	30.99	5662	0.033
Conicity Index	1.2413	0.113	1.34	0.125	0.000
Glycemia (GLY)	6.83	1.55	8417	1.63	0.000
Insulinemia	3231	2303	11,012	3486	0.000
Cholesterol	4.12	1.13	5.35	1.03	0.000
Low-density lipoprotein (LDL-c)	2.01	0.81	2.78	0.69	0.000
Triglycerides/Glucose Index (TGI)	8.9629	0.510	9.6236	0.545	0.000
	Median	Range	Median	Range	
Age at debut	48.5	40	48	36	0.582
Diabetes evolution time	3	15.33	5.5	11.25	0.000
Tobacco exposure time (TET)	0	30	12	32	0.006
Systolic blood pressure	120	60	135	70	0.079
Diastolic blood pressure	80	twenty	90	fifty	0.000
Mean arterial pressure	95	37	103	fifty	0.002
Size	1635	0.38	1.63	0.30	0.780
Waist circumference	90	53	101.5	59	0.000
Visceral adiposity index	1875	6.23	5.26	71.57	0.000
HOMA-IR	0.8	2.41	3705	4.59	0.000
Triglycerides	1.7	2.29	2.7	5.76	0.000
High-density lipoprotein (HDL-c)	1.25	1.2	0.9	1.4	0.000
Triglycerides/HDL-c Index (THI)	1255	3.1	2865	32.8	0.000
Proatherogenic Index	1.5	3.77	3195	12.56	0.000
Castelli Index	3465	3.97	5.89	33.83	0.000
Mean fasting blood glucose	5.85	3.6	7.15	4.8	0.000
Average postprandial blood glucose	12.65	5	14.1	6	0.000
Discriminant model	−8.29075	9.7459	−1.9262	12.9878	0.000

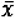
**:** Arithmetic mean; SD: Standard deviation.

**Table 2:** Results of the discriminant analysis.

Variables in the Model	Structure Matrix	Discrimination Coefficients (a_p_)		*p*
		Insulin Resistance		
		No	Yes	
TET (X1)	0.24	−0.186	−0.075	0.000
BMI (X2)	0.28	2457	2257	
GLY (X3)	0.39	1612	2263	
LDL-c (X4)	0.41	−0.943	0.712	
HDL-c (X5)	−0.69	37,235	28,445	
Constant (a_0_)	-	−62,940	−57,662	

TET: Tobacco exposure time; BMI: Body Mass Index; GLY: Glycemia; LDL-c: Low-density lipoprotein; HDL-c: High-density lipoprotein.

**Table 3:** Efficacy of the discriminant model in the detection of patients with insulin resistance.

Parameters	Cut-Off Point	Sensitivity	Specificity	PPV	VPN	Youden’s Index	Area under the Curve	*p*
TET (X1)	1.5	0.68	0.62	0.77	0.51	0.30833	0.66	0.008
BMI (X2)	29.17	0.68	0.78	0.85	0.56	0.46458	0.70	0.001
GLY (X3)	7.95	0.66	0.81	0.86	0.56	0.47916	0.75	0.000
HDL-c (X4)	1025	0.90	0.88	0.94	0.80	0.78958	0.91	0.000
LDL-c (X5)	2.05	0.85	0.56	0.78	0.66	0.41250	0.76	0.000
Discriminant model	−5.4893	0.95	0.90	0.95	0.90	0.85625	0.96	0.000
TGI	8.94	0.95	0.53	0.79	0.85	0.48125	0.81	0.000
THI	1.85	0.91	0.87	0.93	0.84	0.79166	0.93	0.000

TET: Tobacco exposure time; BMI: Body Mass Index; GLY: Glycemia; LDL-c: Low-density lipoprotein; HDL-c: High-density lipoprotein; TGI: Triglycerides/Glucose Index; THI: Triglycerides/HDL-c Index; PPV: Positive predictive value; NPV: Negative predictive value.

## Data Availability

Not applicable.
